# Exploring how mothers with a history of intimate partner violence experienced the COVID-19 pandemic in Ontario, Canada

**DOI:** 10.1371/journal.pgph.0006072

**Published:** 2026-04-10

**Authors:** Kimberley T. Jackson, Emma Butler, Tara Mantler

**Affiliations:** 1 Arthur Labatt Family School of Nursing, Faculty of Health Sciences, The University of Western Ontario, London, Ontario, Canada; 2 School of Health Studies, Faculty of Health Sciences, The University of Western Ontario, London, Ontario, Canada; PLOS: Public Library of Science, UNITED STATES OF AMERICA

## Abstract

In Canada, approximately 44% of women will experience intimate partner violence (IPV) in their lifetime. Given that the majority of women who face IPV are within childbearing years, IPV often co-occurs with being a mother. While research exists on mothering and IPV, a gap in the literature exists with respect to how mothers with histories of IPV experienced the COVID-19 pandemic. This qualitative study addressed this gap using an interpretive description approach underpinned by intersectionality and informed by Ford-Gilboe’s *strengthening capacity to limit intrusion* theory. Twelve, semi-structured virtual interviews were completed with mothers that had experienced IPV greater than 6 months prior to study enrollment. Mothers described experiencing structural violence during the pandemic, impacting their financial stability, access to affordable housing, and access to legal/community supports. Mothers also described the negative impact of the pandemic on their mental health, their difficulty accessing counselling services, and the challenges associated with managing an increased workload. Despite these experiences, many mothers described how their relationship with their child strengthened. Implications of this study include the need for policy changes to address the structural violence experienced by mothers with a history of IPV in the aftermath of the pandemic.

## Introduction

Intimate partner violence (IPV) is a global human rights concern [1], and refers to a pattern of physical, sexual, and/or psychological violence by a current or former intimate partner within the context of coercive control [2]. Though IPV can impact anyone, IPV is a gendered issue disproportionately impacting woman [3], with global estimates of 35% of women experiencing IPV in their lifetime [1]. IPV occurs irrespective of a woman’s age, however, the greatest prevalence is among women of reproductive age [4]. Given that in Canada an estimated 51.1% of women become mothers, for many women, the experience of IPV will often co-occur with mothering [5]. Experiences of IPV can negatively impact parenting for mothers in numerous ways; for example, abusive partners often create environments where mothers are the sole parent, they limit mothers’ access to financial resources, and exert control over mothers’ parenting practices [6,7]. Mothers also have the added challenge of managing their child’s safety, in addition to their own, while in an abusive relationship [6].

Leaving an abusive relationship has additional considerations when children are involved [8]. For example, mothers may have to consider their ability to access domestic abuse support services such as family shelters, or the presence or absence of social supports needed to help with childcare or to afford daycare [8]. Factors such as service accessibility, connections with a mother’s family and social supports, and income impact how a mother supports herself and her children after leaving an abusive relationship [9].

One major factor that has recently impacted service accessibility, connections with family and friends, job security, and income was the COVID-19 pandemic [10,11]. The closure of schools, day care centres, and workplaces meant that parents were required to juggle employment and childcare needs all while working at home [11]. In addition, parents with school-aged children navigated the responsibilities associated with emergency remote learning [11]. The consequences of these public health policies have largely been described as gendered, with women bearing the brunt of the additional household and childcare responsibilities [12]. Furthermore, the closure of businesses and restrictions on in-person gatherings meant employment opportunities for in-person sectors were reduced [10,13,14]. Challenges related to job stability and income security for those working in these in-person, lower-paying sectors continued throughout the duration of the pandemic [14].

Beyond the reality that the COVID-19 pandemic impacted the lives of everyone, it had a significant impact on women who were experiencing IPV. An increased incidence of IPV was reported during the pandemic, with calls to the Canadian Assaulted Women’s Helpline increasing by 64% during a 3-month period in the pandemic compared to pre-pandemic times [15]. This increase in IPV was associated with increased household stress, heightened levels of unemployment, reduced ability for women to access their social networks, and limited access to protective services such as crisis centres and shelters [16,17]. Perpetrators of abuse often used the stay-at-home orders as a means to further coercive control, preventing women experiencing IPV from leaving the house to access needed services [16,18]. As such, the stay-at-home orders and isolation that resulted from the pandemic had the potential to further abuse within this population [19].

A syndemic – defined as the co-occurrence of several health conditions which are often driven and exacerbated by structural and social inequities – results in disproportionate harm to equity-deserving groups [20]. It is important to view COVID-19 as a syndemic, as it shifts the focus away from individual factors, and toward the conditions which cause inequities, thus emphasizing the need for system level changes versus just biomedical responses [21]. Accordingly, research has suggested that women experienced not only a pandemic, but a syndemic during COVID times; this syndemic involved the convergence of both the COVID-19 pandemic and the IPV pandemic, placing disproportionately greater risks on women’s health and wellbeing [22].

The theory of *strengthening capacity to limit intrusion* (SCLI) [9] highlights how service accessibility and connections with family and friends can have great influence on a mother’s ability to position herself and her children for the future [9]. It is often difficult for a new single-parent family to develop stability without the support of services, family, or friends [9]. While it is clear that the COVID-19 pandemic impacted service accessibility, social supports, and financial resources, amongst other things, for single mothers with a history of IPV [10,11,23], research has not directly explored the experiences of mothers with histories of IPV during the COVID-19 pandemic. As such, the purpose of this study was to explore how mothers with a history of IPV experienced the COVID-19 pandemic.

## Methods

### Ethics statement

This sub-study was conducted as part of a larger, arts-informed, qualitative study seeking to understand the experiences of gender-based violence (GBV) among mothers in Ontario, Canada. Ethical approval for this sub-study was obtained from Western University Health Sciences Research Ethics Board (approval #120675).

### Methodology

This study employed an intersectional lens [24] while using interpretive descriptive (ID) methodology [25]. Interpretive description is a qualitative research approach best used when there is a goal of understanding and exploring a clinical phenomenon related to the experiences of a defined population and seeks to find important similarities between individual experiences and identify the underlying meanings and significance behind shared experiences [25]. Though incidences of IPV during the pandemic may have increased within the population as a whole, existing societal power imbalances related to gender and its various intersectional factors, such as class and race, were only worsened during the pandemic [26,27]. Structural violence is described as the social factors (e.g., economic, political, legal, religious, and cultural) which prevent people from reaching their potential [28]. Examples of structural violence may include things such as a lack of employment opportunities, disparate access to goods and services, and inadequate or unaffordable housing [29]. Given this, employing an intersectional lens fits well with research of phenomena where structural violence is at play, as it demonstrates how interconnected systems of power (e.g., racism, sexism, and colonialism) work together to produce predictable and unequal harm. Intersectionality allows the researcher to see beyond a single axis, and to identify the structural conditions which are shaping the experiences of equity deserving groups [22].

The SCLI theory [9] underpinned this study by supporting the development of the research question and study aims, as well as guiding data analysis. SCLI is a feminist theory which illuminates the complex ways that health promotion behaviours are undertaken by single-parent families in the aftermath of experiences of IPV [9]. The theory connects knowledge about health promotion, IPV, and single-parent families to provide a holistic understanding of how single-parent families use their strengths to grow and prosper after leaving an abusive situation [9]. The theory defines the concept of intrusion, which is the undesirable consequences resulting from abuse and its repercussions [9]. Sources of intrusion include harassment from an abuser, health challenges resulting from the abuse, challenges with accessing supports, financial losses or poverty, social isolation, the loss of jobs due to relocation, and the challenges associated with being a single parent [30]. Furthermore, one type of intrusion can be exacerbated by another source of intrusion, with each of these sources of intrusion acting as obstacles for families that are managing the aftermath of an abusive relationship [30].

### Sample

Recruitment for this study ran concurrently with the primary study. Convenience sampling was used to recruit participants for the primary study between 29/04/2022 – 06/12/2022 via advertisements targeting mothers from Ontario, Canada, posted on Kijiji (an online classified service in Canada). To be eligible for inclusion in this sub-study, participants: 1) were 18 years of age, or older; 2) spoke and read English; 3) had access to the internet; 4) self-identified having a past experience of IPV (greater than 6 months ago); 5) identified as a mother; and 6) agreed to being audio-recorded during their one-to-one interview.

### Data collection

Informed consent was obtained by the research assistant (RA) after participants had an opportunity to review the letter of information. Consent was obtained verbally at the beginning of the one-to-one interview and documented on a master spreadsheet. Then, participants completed a semi-structured, one-to-one interview via Zoom with one or two RAs, lasting approximately 60–90 minutes. The RAs were registered nurses in a Master of Nursing graduate studies program who received additional training in trauma- and violence-informed care and qualitative research methods. During the interviews, participants were asked about their experiences during the COVID-19 pandemic, using a semi-structured interview guide. Questions guiding the interview included: 1) How has the COVID-19 pandemic impacted you and your family?; 2) How has the pandemic impacted you as a mother, with your history of experiencing IPV?; 3) Has the pandemic impacted your ability to cope with or process the experience of IPV?; 4) Has the COVID-19 pandemic impacted you or your family’s safety or security?; 5) Has the pandemic impacted your access to social supports in any way?; and 6) Has the COVID-19 pandemic impacted how you access support services?

After each interview was completed, the audio recording was securely transferred to a transcription service where audio files were transcribed verbatim. In line with ID, field notes were taken by the RA throughout each interview to highlight key points, as well as describe emotions, feelings, and thoughts that would not have been captured in the interview transcript [25,31]. All participants were engaged in a post-interview debrief with the RA, where community supports were provided to the participant. Following the interview, participants were emailed a $30 (CAD) Amazon e-gift card as an honorarium for their time.

### Analysis

Inductive, or emergent, coding was then used to detect patterns within the transcripts and reorganize data into a usable format [32,33]. Open and axial coding were used to examine and organizing the data [25] and to identify patterns, key quotes, and thematic ideas [25,31]. Following coding, working with the data was an iterative process involving immersing in the data, stepping away from the data for a period of time, and then re-immersing in the data [25]. Relationships between the various codes were identified, and the data were carefully interpreted and restructured into tentative themes and categories [31]. Multiple thematic iterations were created and revised until consensus was reached by the research team [25]. Lastly, the criteria for data trustworthiness in qualitative research, as outlined by Lincoln and Guba (i.e., confirmability, credibility, dependability, and transferability), [34], were applied in this study. Examples of such strategies to enhance trustworthiness included ongoing reflexivity (journaling/team discussion) to identify and mitigate bias, in addition to data/analyst triangulation using multiple data sources and independent review by each member of the research team. Dependability and transferability were supported via accurate documentation of the study design, data collection and analysis processes, and via the provision of rich, detailed descriptions of the findings.

## Results

Twelve participants were included in the sub-study. Demographic characteristics are reported in Table 1.

**Table 1:** Participant demographic characteristics.

Category	n	%
**Age (years)**		
18-27	0	0
28-37	2	17
38-47	9	75
48+	1	8
**Ethnicity/Cultural Background**		
Canadian	2	17
Mixed Canadian	4	32
Asian-Caribbean	1	8
Jewish-Lithuanian	1	8
French	1	8
Indigenous Canadian	1	8
Mexican	1	8
Latin	1	8
**Marital Status**		
Married, common law, or engaged	4	33
Single	3	42
Divorced or separated	2	17
Other	1	8
**Number of Children**		
1-2	8	67
3-4	3	25
5	1	8
**Living Situation**		
Lives with their child(ren)	5	42
Lives with partner and their child(ren)	4	33
Lives with partner	1	8
Other	1	8
**Living Situation of Children**		
Living with full-time	8	67
Do not live with	2	17
Other	2	17
**Place of Residence**		
Urban	10	83
Rural	2	17
**Education**		
Less than high school	1	8
Some college/university	4	33
College or university degree	6	50
Master’s or doctoral degree	1	8

Analysis revealed four themes highlighting how mothers with a history of IPV experienced the COVID-19 pandemic. These themes included: 1) amplified structural violence; 2) mental health and access to counselling services during COVID-19; 3) loss of caregiving infrastructure and intensification of unpaid labour; and 4) strengthening family relationships.

### Amplified structural violence

Participants discussed numerous ways in which the COVID-19 pandemic exacerbated and/or created sources of structural violence for them, including financial strain secondary to job loss, difficulty securing safe and affordable housing, and difficulty accessing legal services and community supports. One predominant source of structural violence that many mothers experienced was financial strain secondary to job loss. Inequities associated with a high cost of living as well as job loss for individuals in lower-paying sectors during the pandemic created financial challenges for several participants. Participants explained the impact their job loss had on their financial security, and how it led to feelings of uncertainty and stress during the pandemic. This was exemplified by one mother who was in school, investing in education for a career change when the pandemic happened; she worked a full-time job in the entertainment industry to support herself and her child and to afford her schooling after leaving an abusive ex-partner. She noted how “for the last five years I was working in the entertainment industry and so we had a pretty quick shut down as you might … guess with the pandemic”. [P6] She described the challenges she had experienced while managing after job loss:

I went from working fulltime to having no job. There was a lot of uncertainty at that time to when might events get back going. Again, I was already in the process of shifting to a different career… I was sort of thinking how am I even going to afford school because again tuition costs are pretty expensive, that was sort of my planning in terms of saving for school... So yeah, yeah so it did take a financial toll for sure. [P6]

As a single mother, the financial strain that resulted from her job loss during the pandemic had an impact on herself and her child. She was working fulltime to pay for her tuition and support her child, so her job loss during the pandemic had a significant, negative impact on her ability to pay for her schooling.

A second source of structural violence that mothers experienced was difficulties accessing affordable housing. A priority for participants during the COVID-19 pandemic was ensuring access to safe and affordable housing for themselves and their children. However, the structural violence associated with poverty, income, and job loss during the pandemic made it challenging for some participants to obtain affordable housing. One mother described the challenges associated with housing relocation during the pandemic. Her experience of structural violence centered around the lack of safe housing options for mothers that relocated to escape abusive relationships. She had just left an abusive partner a few days before the pandemic and was accessing emergency housing to keep herself and her children safe:

We got lucky in the sense that we had to do emergency housing, like in a co-op, because my rent at the time was $2,000 for a one plus one bedroom. So, yeah. And obviously, not sustainable, I was on nothing. And I had to go on ODSP [disability benefits]. So we moved to a co-op … I have never seen co-ops, I have never been around them, I’ve never experienced that life. We moved the day before lockdown. To a city two hours away where I knew nobody, to a co-op home. My children were one and two by the time we moved to the co-op. There were no playgrounds, no parks …the problem was the co-op was a not safe place… There were times I didn’t have furniture. They couldn’t send furniture until the furniture banks opened again after the lockdown. I couldn’t get anything, couldn’t go to the store… I erased a large part of that time from my mind. [P5]

As the pandemic began, this participant was managing alone in an unsafe housing situation without access to necessary resources. As a result of her relocation and low income coming from the Ontario Disability Support Program (ODSP), she was not able to afford a more suitable place for her family.

Difficulty accessing necessary legal services was another way that mothers with a history of IPV experienced structural violence during the COVID-19 pandemic. One participant, who visited her daughter at a visiting centre (provincial sites for supervised visits with children) prior to the pandemic, described her challenges with accessing legal services. This participant’s ex-partner, who had custody of her child, restricted the participant’s visiting rights during the pandemic; he opted out of using virtual communication platforms that were put in place by the visiting centre when in-person visiting could no longer occur between the participant and her daughter. Though the participant considered taking legal action against her ex-partner so that she could regain visiting rights with her daughter, she described how it felt like a pointless endeavor given the state of the legal system:

Throughout the whole entire thing I had like a couple people tell me oh, well you can go to court to like make the judge, you know make your ex let you see the kid more often. But every other parent that I talked to who was going to court like during that whole time, like family court is usually slow and it [the pandemic] made it like twice as long. So, you’d be lucky to get a court date within nine months and usually at the first three court dates all they say is come back later, come back later, come back later. So, by the time you start a court date you’re not going to get a result for like 18 months. It’s like, why bother? [P1]

For this mother, her ex-partner’s restriction of her visiting rights and her inability to access timely legal services prevented her from spending time with her child.

### Mental health and access to counselling services during COVID-19

An important part of mothers’ experiences during the COVID-19 pandemic involved the impact of the pandemic on their mental health. Several participants described experiences of poor mental health during the pandemic, which impacted their role as a mother. Participants described their distressed emotional state including terms such as “extreme distress” [P5], “it was the worst” [P7], “overwhelming” [P12], and “really stressful” [P1]. One participant explicitly described how her emotional state during the pandemic impacted her role as a mother. She had just left an abusive relationship and was dealing with several challenges because of relocation and the closure of services during the pandemic:

I was not able to be the mom I wanted to be. Period. The mom that I wish I could have been. I had no money. There were days I didn’t eat. And they didn’t understand why I cried so much, why I yelled so much on the phone or in my room, or why mommy was sad, why mommy was angry. [P5]

This participant also noted the compounding nature of the stressors and how the COVID-19 pandemic exacerbated an already untenable situation, stating,

You cannot take that much and keep going and keep going…It’s like you accrue trauma, it does not get sorted, you don’t get the support you need, you just keep accumulating it. COVID was awful. It was an awful addition to an already awful situation”. [P5]

Participants that accessed counselling services during the pandemic identified concerns with their ability to access such services in a private and confidential manner through the virtual care platforms at home. This made it all the more difficult for participants to cope with their past experiences of IPV, as well as any ongoing abuse that they were experiencing. When discussing her experience accessing virtual care while her son was at home, one participant said:

It was safe during the day and I was trying to time it when my son would be in an online class at the same time just because I was thinking I don’t really want him to hear maybe perhaps all the things that are going on, that kind of stuff. So I remember yeah, I was trying to time it that way, trying to kind of talk in a quieter voice or make sure maybe there was some music or something going on downstairs so he couldn’t necessarily hear what was going on upstairs. Yeah, definitely that was another barrier too. [P6]

The reduced ability to access counselling services in a way that was seen as effective and private impacted the ability of these participants to work through, and heal from, past trauma. As women who had experienced IPV, it was important for them to access counselling services; however, as single mothers, they struggled to find the privacy that they needed to have these conversations.

Despite these challenges, mothers often engaged in creative problem solving around these concerns; for example, turning on music, talking in a hushed voice, and giving children tasks to distract them while they were in session with their counsellors. However, privacy concerns were still prevalent for mothers in this study despite these strategies.

### Loss of caregiving infrastructure and intensification of unpaid labour

Participants who had children living at home with them during the pandemic experienced an increased workload. For several participants, this increased workload was due to the loss of caregiving options. Many parents were required to care for their children at home while simultaneously working and managing a household. One participant, who had left her abusive partner a few years prior to the pandemic, was used to having her child in school during the daytime. When discussing the impact of the pandemic on her workload, she said:

When school had started for him, it was the first year – it started in September and then the pandemic came in March, so it was the first time that I actually had relief care for my child, because it was just us together before. So that was tricky, because I was just starting to get used to having someone else – somewhere else to take him to, and then isolating – very isolating. [P3]

For mothers that had children attending daycare prior to the pandemic, the closure of daycare centers had a big impact on their workload and livelihood. One mother that had left an abusive partner a few years prior to the pandemic discussed the impact that the closure of daycare centers had on herself and her daughter:

And in terms of COVID, it’s been a bit hard because there’s basically no daycare access, especially during the height of the pandemic. So, she was just kind of like staying with me 20 – almost 24-7. Yeah. It’s been pretty tough…So kind of trying to juggle between taking care of her 24-7 and also trying to get enough income. So, just trying to balance everything. [P2]

For this participant, who was a single mother and sole provider for her child, the challenges associated with balancing childcare and work were stressful. Her role as a mother was impacted, as she was taking on additional childcare responsibilities and experiencing a loss in income during the pandemic.

### Strengthening family relationships

After leaving an abusive relationship, mothers went through a process of redefining their family structure. With the layering of the pandemic context, mothers with a history of IPV described how this process of developing new familial relationships sometimes created positive change for them. One participant described her family, saying “as a family I think we got time to know each other more…We’re closer than we used to be.” [P9] The changing nature of the relationships between mothers and their children was something that several participants described. One participant, who left an abusive partner and became a single mother just prior to the pandemic, said:

I actually felt closer to her just because we spent so much time together and I think we kind of bonded a lot as well. Not to say that we weren’t bonding before. But yeah. I think I felt closer to her. And especially just watching her grow day to day. And now that she’s kind of able to kind of speak a lot compared to kind of back when she was an infant. Yeah. So it feels like she’s like a best friend now. [P2]

Another mother, who was also a single parent, echoed this evolving relationship she had with her child, and the way that the pandemic shaped the way that she mothered. Specifically, she discussed how the increased amount of time she spent with her child made her feel like she was able to open up to him more:

I think just by virtue of getting to spend a lot more time with my son, I used to sort of work fulltime and be outside of the house and he’d be outside of the house. I think we got to spend a lot more time together…when he was here we were together all day long, so we spent a lot of time together. And yeah, so I think I really got to know maybe different parts of him and really appreciate different things about him too…there’re actually ways that I was able to like in a, sort of in an age-appropriate way, kind of open up to him a bit more about sort of me and who I am [P6].

The pandemic positively impacted the relationship that many mothers had with their children. Mothers described a change in their parenting practices, including spending more time outside with their family and opening up to their children in age-appropriate ways. This reflects another way that the pandemic shaped the experiences of mothers with a history of IPV.

## Discussion

The purpose of this study was to explore how mothers with a history of IPV experienced the COVID-19 pandemic. Major findings from this study included mothers’ experiences of structural violence during the pandemic, mental health challenges that arose, and difficulties associated with accessing private counselling supports. Furthermore, challenges with balancing work, childcare, and emergency remote learning were described, as well as the strengthening of family relationships that occurred during the pandemic.

Findings from this study highlighted the structural violence that mothers with a history of IPV experienced during the COVID-19 pandemic. For example, mothers described experiencing financial strain secondary to job loss during the pandemic. Existing literature supports these findings, noting that job loss, financial insecurity, and housing challenges existed for single mothers with a history of IPV during the pandemic [35]. Existing literature also discusses how financial obstacles are common for women that leave abusive relationships [7,9]. This study built on existing research to describe the financial challenges that arose for mothers with a history of IPV during the pandemic, specifically within a Canadian context.

Findings from this study underscored the trauma and distress that mothers with a history of IPV experienced during the pandemic. Existing research highlights the importance of social supports and community services for mothers that have left an abusive relationship [36,37]. This study demonstrated the negative impact that the closure of these services during the pandemic had on the wellbeing of mothers that had left an abusive relationship. Furthermore, this study reports the phenomenon of compounding trauma after leaving an abusive partner in this context, specifically how the pandemic contributed to a “layering” of trauma experienced by women and their children during the pandemic. Existing literature has described the negative impact that IPV can have on the mental health of mothers, and how mental health challenges can continue even after leaving an abusive relationship [38]. This study adds to the existing literature by describing how the pandemic was yet another challenge in the layered trauma that women with a history of IPV often endure.

Given the impact of the pandemic on the mental health of participants, as well as their past experiences of IPV, participants had a desire to access counselling supports. However, participants described challenges with accessing these supports in a private and confidential manner while at home with their children – finding it challenging to find time alone to access these services. This finding supports existing research on the challenges of accessing counselling services during the pandemic. For example, a Canadian study of women experiencing IPV during the pandemic [39] found that women who had left abusive relationships faced challenges accessing formal support services, especially when their children were in close proximity. The current study adds new insights into some of the problem-solving strategies that mothers engaged in to maintain a semblance of privacy when talking with a counsellor.

In addition to privacy challenges at home, mothers in this study also experienced challenges with balancing their work, family, and emergency remote learning responsibilities. These challenges were described primarily by single mothers, who were often balancing these tasks without partner support. Existing research on mothering during the COVID-19 pandemic supports this finding; with mothers in single-adult households reporting less childcare support during the pandemic compared to mothers having other adults in the home [40]. Single mothers without partners or other support also felt that their productivity was impacted while working from home, compared to mothers with other adults at home [40].

Though mothers with a history of IPV experienced numerous additional challenges as a result of the COVID-19 pandemic, mothers often described a process of strengthening family relationships that occurred during this time. This finding is supported by existing literature on the impact of the pandemic on mothers with experiences of IPV. Similar to this study, existing literature reports how the pandemic allowed mothers to spend more time with their children [41]. However, for participants in this study it was more than just time together, the pandemic afforded them the opportunity for evolution of the relationship between mother and child(ren). Of note, this study added insight into the evolving relationships that happened during these moments of family bonding in the pandemic, with participants discussing opening up to their children in new ways and feeling like their connection with their children changed for the better during the pandemic.

Prior Canadian research has highlighted how COVID-19 resulted in greater isolation, barriers to services, and other socioeconomic vulnerabilities among women with experience of IPV [42]. However, these studies centred service barriers and providers, versus our study which centred the lived experiences of mothers experiencing IPV. In addition, reviews have drawn to light gendered, unpaid care burdens and their intersections with poverty, housing instability, and challenges with mental health [43]. We have extended this knowledge by integrating mothers’ first-hand experiences of the amplification of structural violence, impacts on mental health, and caregiving overload resulting from the COVID-19 pandemic in a Canadian context.

The four themes identified in this study—amplified structural violence; mental health and access to counselling services during COVID-19; loss of caregiving infrastructure and intensification of unpaid labour; and strengthening family relationships—mapped closely onto the core principles of the SCLI theory [9]. For example, participants reported amplified experiences of financial strain, housing insecurity, impeded legal processes, and lack of access to community and mental health services as a result of the COVID-19 pandemic. These experiences demonstrate how structural violence was at play, and how the sources of intrusion were exacerbated, thus constraining mothers’ capacity to safeguard their health and to care for their children as desired. This is in line with SCLI’s conceptualization of intrusion as cumulative and mutually reinforcing [9]. In addition, intrusions experienced by mothers were further compounded by the increase in caregiving demands that came along with trying to balance their paid work, childcare, and emergency remote learning, all of which led to emotional distress and less opportunities for recovery and healing. Lastly, processes of family empowerment and resilience described in SCLI [9] was evident in our findings, highlighted by participants’ descriptions of adaptive coping, creative problem-solving, and strengthened parent–child relationships.

The findings from this study show interconnectivity among the themes. For example, the amplification of structural violence evidenced through financial insecurity, precarious housing, impeded legal processes, and lack of access to services were chronic stressors which served to undermine mental health. This is consistent with other evidence linking structural inequities and IPV to deleterious psychological outcomes [44]. Furthermore, stressors were also intensified by challenges with childcare (e.g., school/daycare closures) which placed additional responsibilities onto mothers and subsequent emotional exhaustion which compounded the effects of trauma and inhibited recovery [45]. Conversely, participant descriptions of strengthened parent–child relationships showed a critical resilience mechanism, which often enabled mothers to derive meaning, support, and emotional grounding despite their challenges [46]. Together, these findings suggest that mental health impacts cannot be separated apart from structural and caregiving contexts, and that relational strengthening can be an adaptive response to intrusion and an opportunity to mitigate the harms of intersecting crises.

### Limitations

The findings from this study must be examined within the context of its potential limitations. First, the findings of this study are limited to the experiences of mothers from Ontario, Canada; as such, the findings are only relevant to this population. Second, the recruitment for this study took place solely on Kijiji, limiting the pool of participants to those that access this site; further, convenience sampling (via Kijiji) may create significant bias, thus limiting representativeness. This recruitment method also relied on participants’ self-selection to engage in the research study, as opposed to researchers seeking out participants within a defined population. Future studies would benefit from using a more comprehensive recruitment approach to target audiences in diverse online and in-person platforms. In addition, data were not collected specifically around the length of time that had lapsed since leaving an abusive relationship. Not having these data may have limited our contextualization of the experiences shared by women; for example, experiences of those who had recently left an abusive relationship may differ from those who left their abusive relationship years prior to the study. Finally, the sample was limited to the 12 participants who had experienced GBV who specifically identified as having experienced IPV. While the data obtained were rich and sufficient to address the research question, it is possible that if the sample size was larger there may have been other findings that might differ with what was presented here.

### Implications for policy and future research

This study has important implications for public health policy and future research. For example, the inequitable impact of the pandemic on mothers who had experienced IPV was made apparent in this study. In order to address the impact of the pandemic on this population and to prepare for future public health crises of a similar nature, policy changes are necessary. In a recent publication discussing the COVID-19 pandemic, intersectionality, and structural inequity, it was described how public health officials have an obligation to center and “address the health, economic and social needs of those who bear the intersectional brunt of structural inequality” [47]. As the COVID-19 pandemic ends, Bowleg [47] noted how it is unacceptable to maintain the status quo, as the status quo privileges certain members of the population and disadvantages others. This sentiment resonates with the findings of this study. For example, this study highlighted inequities that were experienced by mothers of lower socioeconomic status who had experienced IPV. Policy changes are necessary to address the compounding challenges mothers faced in order to mitigate the financial, housing, and employment challenges associated with leaving an abusive relationship. One such example would be to ensure that court and legal services continue should another pandemic occur.

In addition, findings from this study underscored that it was critically important that a living wage was obtainable to get through such public health crises, as the challenges with obtaining financial security and safe housing for women with experiences of IPV in this study were only exacerbated during the pandemic. This demonstrates how policy changes are essential to provide women with the resources that they need to support their families and grow in the aftermath of an abusive relationship, and in the aftermath of the pandemic. The Registered Nurses’ Association of Ontario (RNAO) [48] recommends enhanced collaboration between the various health and social service industries; the best practice guideline outlines the importance of health systems, social service systems (housing, employment), justice systems, and education systems working together, with the support of individuals that have lived experiences. Collaborations such as this would be an important way to support women that have experienced IPV in the aftermath of the pandemic.

## Conclusion

This study contributes to the understanding of how mothers with a history of IPV experienced the COVID-19 pandemic. Through this study, it became apparent that mothers with a history of IPV faced numerous challenges, and sources of structural violence during the height of the COVID-19 pandemic. Challenges with securing safe and affordable housing, job loss, and difficulty accessing legal and community resources were some of the primary ways that participants experienced structural violence. In addition, challenging experiences faced during the pandemic, such as difficulties with accessing formal supports, and efforts to balance their increased workload, compounded the negative impacts of the pandemic on this population. Despite these challenges, mothers worked to strengthen family relationships and continue to grow in a positive way. In order to support mothers in a post- COVID-19 world, and to prepare for future crises such as this, practice and policy changes need to occur to ensure equitable access to housing, food, and other resources.
